# Psychometric properties of a questionnaire (HEMO‐FISS‐QoL) to evaluate the burden associated with haemorrhoidal disease and anal fissures

**DOI:** 10.1111/codi.14393

**Published:** 2018-09-21

**Authors:** L. Abramowitz, D. Bouchard, L. Siproudhis, M. Trompette, H. Pillant, C. Bord, C. A. Senejoux, C. Favreau‐Weltzer, G. Berdeaux, A. Zkik

**Affiliations:** ^1^ AP‐HP Proctologie Médico Chirurgicale du Service de Gastroentérologie CHU Bichat Paris France; ^2^ Clinique Blomet Ramsay GDS GREP (Groupe de Recherche en Proctologie de la SNFCP) Paris France; ^3^ Service de Proctologie Hôpital Bagatelle Talence France; ^4^ Service de Gastroentérologie et Groupe Imphy CIC1414 CHU Pontchaillou Rennes France; ^5^ Service de Proctologie Groupe Hospitalier Saint Joseph Paris France; ^6^ Polyclinique de Saint Roch Montpellier France; ^7^ CHP de Saint‐Grégoire Rennes France; ^8^ Berdeaux Consulting SAS Chambourcy France; ^9^ Pierre Fabre SA Boulogne‐Billancourt France

**Keywords:** Haemorrhoids, anal fissures, quality of life, patient‐related outcomes

## Abstract

**Aim:**

Current questionnaires designed to evaluate the burden of haemorrhoidal disease ignore symptoms such as bleeding, pain and itching. A specific questionnaire is needed to evaluate the global impact of anal disorders on patients’ daily lives.

**Method:**

We developed a questionnaire (HEMO‐FISS‐QoL) to assess the symptom burden of anal disorders and administered it to 256 patients (mean age 46.2 years; men 60.4%) with haemorrhoidal disease (67.2%), anal fissure (29.3%) or both (3.5%). Psychometric properties were evaluated by testing the acceptability, construct validity and reliability of the questionnaire. Principal components and multi‐trait analyses were used to identify dimensions and to assess construct validity. Backward Cronbach alpha curves and a graded response model were used to reduce the number of items and modalities. External validity was evaluated against SF‐12 and the Psychological Global Well‐Being Index (PGWBI) using Spearman's correlation coefficient.

**Results:**

Principal component analysis defined four dimensions: physical disorders, psychology, defaecation and sexuality. The number of questions was reduced from 38 to 23. The HEMO‐FISS‐QoL scores correlated well with those of the SF‐12 and PGWBI (*P *<* *0.001). Cronbach's coefficients (all > 0.7) reflected good internal reliability of the different dimensions. The total score increased with the severity of the anal disorders and with their consequences (days off work and personal spending related to the disease).

**Conclusion:**

The HEMO‐FISS‐QoL questionnaire reliably evaluates the global impact of haemorrhoids and anal fissures on patients’ daily lives. This simple tool may prove useful for treatment evaluation in clinical trials and daily practice.

**What does this paper add to the literature?**

This study validates the first tool developed to evaluate the overall impact of haemorrhoidal disease and anal fissures on patients’ quality of life. The HEMO‐FISS‐QoL questionnaire should prove useful for research and routine clinical purposes.

## Introduction

An anal fissure is an ulcer arising in the squamous epithelium just distal to the mucocutaneous junction 1. Systematic screening showed anal fissures in 15% of 259 women 2 months after delivery 2. Anal fissures usually manifest suddenly, with intense anal pain triggered by defaecation, and may be accompanied by anal bleeding and itching. Most patients recover spontaneously or after medical 3 or surgical treatment 4, 5, but relapses are frequent. Despite the frequency of anal fissures, there are no severity rating scales. Pain can be quantified with a visual analogue scale (VAS), but its frequency and intensity are highly variable from one patient to another. Bleeding and itching are very difficult to evaluate. It would be useful to have means of measuring the global impact of anal fissures on patients’ daily lives.

Haemorrhoidal disease is a common chronic disorder 6. Pregnant women are particularly at risk, with external thrombosis being reported in 9% and 20% of women during pregnancy and after delivery, respectively 2. Most cases of haemorrhoidal disease occur between the ages of 45 and 65, but the entire population is at risk 7. In the United States, at least 50% of the population will develop symptomatic haemorrhoids during their lifetime 6. In an Austrian prospective study, the prevalence of haemorrhoids in the general adult population was about 40% 7. The main symptoms are bleeding, swelling, itching, soiling and pain. Internal haemorrhoids can give rise to prolapse and bleeding, and external haemorrhoids to thrombosis. Goligher's classification is the only available clinical tool for evaluating haemorrhoidal disease before treatment 8, but it only assesses haemorrhoidal prolapse and not the severity of bleeding, pain, swelling or itching. Thus, existing tools to guide the choice of treatment encompass only part of the complexity of the pathology 9. The global consequences of haemorrhoidal disease are difficult to assess, including its impact on patients’ daily lives 10.

As haemorrhoidal disease and anal fissures share similar symptoms, we have developed and validated a specific questionnaire (HEMO‐FISS‐QoL) designed to evaluate their impact on patients’ daily lives.

## Method

### Objective and study design

The study was performed according to French law: an ethical committee was not required in the case of a non‐interventional study where nominative information was not collected. Patients were orally informed of the purpose of the interviews.

Our objective was to develop and validate a patient self‐completed questionnaire covering both haemorrhoids and anal fissures that would be sufficiently short and simple for use in research and daily practice. The questionnaire, named HEMO‐FISS‐QoL (HF‐QoL), was developed in collaboration with clinicians specializing in the treatment of haemorrhoidal disease and anal fissures (Groupe de Recherche en Proctologie de la Société Nationale Française de Colo‐Proctologie, GREP).

The present paper focuses on the psychometric properties of a patient self‐completed questionnaire covering both haemorrhoids and anal fissures and designed to evaluate their impact on patients’ daily lives. The development of this questionnaire followed the steps reported below.

A preliminary conceptual framework (CF) was developed using a systematic literature review along with an initial list of questions derived from already existing patient reported outcome aimed at identifying concepts and domains of interest for patients suffering from haemorrhoids and anal fissures.

Semi‐structured patient interviews were conducted on patients suffering from haemorrhoids and anal fissures: after the first 5–10 interviews the remainder were conducted by several researchers at a different site. Participants’ responses were reported verbatim then analysed using qualitative content analyses to understand how patients suffering from haemorrhoids and anal fissures perceived the disease burden and its impact on their daily lives; these responses allowed us to generate items for the questionnaire.

The CF was updated following a meeting with clinical experts from the GREP. This meeting was set up to review and discuss the preliminary conceptual framework and to capture the insights of clinical experts. The clinical experts comprised a proctologist, a gastroenterologist and a surgeon, and they provided their feedback by describing their experiences and revising the preliminary conceptual model.

Cognitive debriefing interviews with patients (*n *=* *5) were organized to review and discuss the preliminary version of the questionnaire in terms of acceptability, comprehensiveness and consistent interpretation.

Based on the information thus collected, a list of 38 questions was compiled with the clinical experts. A cognitive debriefing was conducted, and saturation was assessed on administration to the first patients. All the answers were expressed on a Lickert scale, ranging from 1 (‘never’) to 6 (‘always’). Patients unconcerned by the question recorded a score of 7 (‘not applicable’).

The questionnaire was developed in French, then translated into English using forward translation, reconciliation, back translation and review and cognitive debriefing in the English population of the interest.

The questionnaire was proposed to consecutive patients consulting senior investigating proctologists for haemorrhoids or anal fissures. The psychometric properties of the questionnaire were evaluated by testing its acceptability, construct validity and reliability (see below).

All statistical analyses used SAS software version 9.4 (SAS Institute, Cary, North Carolina, USA).

### Acceptability

The acceptability of the questionnaire was evaluated by considering the proportion of missing values, ‘not applicable’ answers (score 7), and the floor and ceiling effects for each question. The ‘not applicable’ modality was evaluated by using multiple correspondence factor analysis, comparing profiles of ‘concerned’ and ‘unconcerned’ answers.

### Construct validity

Assessment of the construct validity of the questionnaire involved five main steps and a series of complementary analyses.

First, an exploratory principal component analysis (PCA) was conducted, using Varimax rotation to identify the different dimensions of the questionnaire. Second, the unidimensionality of each previously defined dimension was evaluated using backward Cronbach alpha curves (BCAC). Third, a graded response model (GRM) 11, 12, 13 was used to deal with item and modality redundancies.

These methods were used to identify redundant questions in a given dimension and to minimize the number of answers to a given question, in order to obtain the shortest possible questionnaire without losing information.

Fourth, the convergent and divergent validity of the retained items was evaluated by multi‐trait analysis (MTA) in order to confirm the internal validity of the questionnaire. This method measures the relationship between each item and its corresponding dimension, as well as the other dimensions, in order to verify that each item correlates strongly with its own dimension (Pearson coefficient ≥ 0.4) and weakly with the other dimensions.

### External validity

External validity was assessed using SF‐12 14 and the Psychological General Well‐Being Index (PGWBI) 15, 16. The PGWBI is aimed at measuring self‐representations of intrapersonal affective or emotional states reflecting a sense of subjective well‐being or distress. SF‐12, a shorter form of SF‐36, was developed to study the physical functional and mental aspects of patients with chronic conditions.

Haemorrhoidal diseases and anal fissures become chronic in the most severely affected patients with little or no time between crises. As such, the impact of these diseases is on both physical functioning and the emotional aspect (pain, stress, social isolation, etc.) All these dimensions are properly captured by PGWBI and SF‐12. We expected correlations between some HEMO‐FISS dimensions and the above generic scales. Then, as HEMO‐FISS is more specific, with dedicated questions, we expected it to have more sensitivity.

Published algorithms were used to calculate the dimensions of SF‐12 and PGWBI. Spearman correlation coefficients between SF‐12 14, PGWBI 15, 16 and HEMO‐FISS were computed.

### Reliability

Reliability was evaluated by calculating the Cronbach alpha coefficients (CACs) for each dimension. Values above 0.7 were considered to show good internal consistency and reliability 17.

### Scoring

The final version of the questionnaire is composed of 23 items and six modalities, distributed in four dimensions: physical disorders (eleven items), psychology (seven items), defaecation (three items) and sexuality (two items). The six modalities are never (score 1), rarely (2), regularly (3), very often (4), always (5) and not applicable (6). An end user algorithm support is available as Appendix S1.

A score is calculated for each dimension, provided that fewer than 50% of the items are missing (‘not applicable’ items are not considered to be missing data); if 50% or more of the items in a given dimension are missing, a score cannot be calculated and is considered to be missing.

When the score is calculable (fewer than 50% of the items are missing), the score of each dimension is the mean of the items. If missing data are present, specific rules apply for the calculation of the score, as follows:

First rule: if items scored from 1 to 5 (i.e. from ‘never’ to ‘always’) are present in a dimension, the ‘not applicable’ items as well as the missing data are replaced with the mean score of the items completed by the patient (in other words, the score of the dimension is the mean of the items scored from 1 to 5).
Second rule: if zero items are scored from 1 to 5 (meaning that only ‘not applicable’ items or both ‘not applicable’ and missing items are present in the dimension), then the score of the dimension is replaced with the mean score of the study population for this dimension.

The score of each dimension for each patient ranges from 0 to 100 after transformation, according to the following formula:

$\text{transformed score}\left(\frac{\text{patient raw score ‐ lowest possible score}}{\text{highest possible score ‐ lowest possible score}}\right)\times 100.$

The ‘highest possible score’ of each dimension in the formula is 5 (‘always’ for all items) and the ‘lowest possible score’ is 1 (‘never’ for all items). A high score corresponds to a high symptom burden.

A total score is calculated if the scores of the four dimensions are themselves calculable (the total score is not calculated if the score for one dimension is missing). The total score is the mean of the scores of all items (note that the total score is not the mean of the four scores).

The scoring can be simplified in daily clinical practice for a given patient. A score is calculated for each dimension provided that fewer than 50% of the items are missing (‘not applicable’ answers are counted as not missing). The score of each dimension is the mean of the items scored from 1 to 5 (not taking into account the ‘not applicable’ answers). The same rules are applied for the computation of the total score which is calculated if the four dimensions are themselves calculable.

## Results

### Study population

A total of 256 patients were included in the study (Table 1). Their mean (SD) age was 46.2 (13.9) years, 60.4% were men, and more than half were aged over 40 years. They consulted for haemorrhoidal disease in 67.2% of cases and for anal fissure in 29.3% of cases (3.5% had both disorders). Prolapse was present in 79.0% of patients with haemorrhoidal disease (Grade I, 7.7%; II, 37.1%; III, 44.1%; IV, 11.2%), thrombosis in 24.9% (external and circular in 60.0% of cases) and anal bleeding in 58.0%. It was the first consultation in most cases (64.8%). The mean disease duration was 6.2 years. Most patients were currently in crisis (74.9%). A treatment was prescribed to 98.8% of patients.

**Table 1:** Patient characteristics

Characteristics	*n *=* *256
Gender, *n* (%)	*n* = 255
Men	154 (60.4)
Women	101 (39.6)
Age (years)	*n = 231*
Mean (SD)	46.2 (13.9)
Classes, *n* (%)	
<30	28 (12.1)
30–39	54 (23.4)
40–49	57 (24.7)
50–59	48 (20.8)
≥60	44 (19.1)
Type of disease, *n* (%)	*n* = 256
Haemorrhoidal disease	172 (67.2)
Prolapse^a^	143/181 (79.0)
Thrombosis^b^	45/181 (24.9)
Anal bleeding	105/181 (58.0)
Anal fissure	75 (29.3)
Acute	28/84 (33.3)
Chronic	56/84 (66.7)
Both disorders	9 (3.5)
First consultation, *n* (%)	*n* = 247
No	87 (35.2)
Yes	160 (64.8)
Treatment prescription, *n* (%)	*n* = 252
No	3 (1.2)
Yes	249 (98.8)
Previous treatments, *n* (%)	*n* = 249
Medical	108 (43.4)
Instrumental	98 (39.4)
Surgical	48 (17.3)
Disease duration (years)	*N = 223*
Mean (SD)	6.2 (8.3)
Median	3
Currently in acute crisis, *n* (%)	*n* = 255
No	64 (25.1)
Yes	191 (74.9)
Crisis duration (months)	*n* = 94
Mean (SD)	1.1 (1.7)
Median	0.44
Range	0.03–8
Number of previous episodes, *n* (%)	*n* = 248
0	64 (25.8)
1	39 (15.7)
> 1	145 (58.5)

a Grade I, *n *=* *11 (7.7%); Grade II, *n *=* *53 (37.1%); Grade III, *n *=* *63 (44.1%); Grade IV, *n *=* *16 (11.2%).

b External, *n *=* *9 (20%); internal single (or small number), *n *=* *1 (2%); circular external and single (or small number), *n *=* *1 (2%); external and circular, *n *=* *27 (60%); external and internal and circular, *n *=* *3 (7%); external and internal, *n *=* *1 (2%); internal and single (or small number), *n *=* *1 (2%).

Global pain and current pain were estimated with a mean of 2.8 for both, on a VAS scale from 0 (no pain) to 10 (unbearable pain): global pain and current pain scores were significantly higher in patients with anal fissure, either alone or associated with other anal symptoms (Table 2).

**Table 2:** Pain evaluation (visual analogue score, VAS) according to clinical characteristics

Characteristics	*n *=* *256	*P*‐value
All patients		
Global pain^a^	*n* = 256	
Haemorrhoidal disease	1.9 (2.5)	<0.0001^a^
Anal fissure	4.5 (2.5)	
Both	5.3 (2.8)	
Total	2.8 (2.8)	
Current pain^a^	*n* = 244	
Haemorrhoidal disease	2.0 (2.5)	<0.0001^a^
Anal fissure	4.3 (2.4)	
Both	4.6 (3.1)	
Total	2.8 (2.7)	
Patients with only anal fissure		
Global pain	*n* = 75	
Acute	4.7 (2.3)	0.628^b^
Chronic	4.4 (2.6)	
Current pain	*n* = 72	
Acute	4.9 (2.1)	0.1033^b^
Chronic	3.9 (2.4)	

Results are given as mean (SD) of VAS scores from 0 (no pain) to 10 (unbearable pain).

a Kruskal–Wallis test.

b Student's *t*‐test.

### Acceptability

The HF‐QoL questionnaire was well accepted, with rates of missing values below 4% for all items, and no floor or ceiling effect. As multiple correspondence factor analysis showed no major association between the ‘not applicable’ answer and the other modalities, it was agreed to replace missing data and ‘not applicable’ answers by the mean of available scores for each item at the individual patient level.

### Construct validity

After a preliminary comparison of responses from patients with haemorrhoids and those with anal fissures, the questionnaire was considered valid to evaluate the impact of both disorders on quality of life. Indeed, all item scores increased with the clinical burden and were higher for anal fissures than for haemorrhoidal disease, except for the item ‘I'm worried about staining my clothes or the armchair’, where the burden was higher for haemorrhoidal disease. It was thus decided to exclude this item and to construct a single questionnaire with 37 items for both haemorrhoids and anal fissure.

The study sample size was adequate for factor analysis (Kaiser–Meyer–Olkin index = 0.927) 18. PCA identified four dimensions: physical disorders, psychology, daefecation and sexuality. The results of PCA are shown in Table 3. Eight items were discarded after PCA (Table 4).

**Table 3:** Results of principal components analysis after Varimax rotation

Items	Factor 1	Factor 2	Factor 3	Factor 4
I am uncomfortable when doing household chores/tidying up/handiwork	**0.85175**	0.12949	0.13155	0.13435
I am uncomfortable while walking	**0.81459**	0.08507	0.11099	0.19097
I experience discomfort when I climb the stairs	**0.81031**	0.06866	0.03431	0.16762
I am uncomfortable when I play sports	**0.80111**	0.09404	0.11559	0.11755
It is uncomfortable to remain standing	**0.75014**	0.15846	0.17991	0.17791
It is uncomfortable to remain seated	**0.70347**	0.14913	0.29729	0.10331
I do fewer things than I would want to do	**0.69942**	0.32134	0.26373	0.15926
I find it difficult to do my work well	**0.66259**	0.29241	0.31586	0.09763
Driving a vehicle is difficult	**0.62937**	0.14382	0.29689	0.07936
I have to change clothes regularly or use a special type of clothing	**0.57974**	0.22597	‐0.05484	0.35002
I avoid going out (travel, leisure, friends, …)	**0.56468**	0.42506	0.22631	0.15554
Riding a two‐wheeled vehicle or bicycle is difficult	**0.54867**	0.21163	0.23931	0.05712
My family life is disrupted	**0.50690**	0.42013	0.26423	0.33411
I feel tired and exhausted	**0.45340**	0.44842	0.22264	0.22106
I have to wear protection	**0.34269**	0.20182	‐0.14761	0.32642
I feel as if nobody understands my situation	0.13858	**0.71776**	0.14351	0.19402
I feel as if I am different from others	0.25451	**0.70994**	0.17395	0.13256
I feel ashamed	0.16839	**0.65136**	0.05340	0.17862
I believe that my illness is incurable	0.07962	**0.64632**	0.28565	0.04371
My symptoms make me fear I have cancer (or a serious disease)	−0.00700	**0.62244**	0.13698	‐0.02103
I am uncomfortable in my own body	0.30604	**0.59654**	0.25923	0.30020
I get less out of life	0.39052	**0.55385**	0.27597	0.33177
I feel uncomfortable with people around me	0.34570	**0.54138**	0.10313	0.31689
Taking care of my children is difficult	0.33733	**0.47226**	0.16068	0.18260
The pain, blood and lumps worry me	0.15937	**0.45407**	0.40542	0.08320
I find it hard to get to sleep	0.37057	**0.41580**	0.14871	0.28301
I dread bowel movements	0.21866	0.19183	**0.81676**	0.17030
I am uncomfortable during bowel movements	0.18359	0.12851	**0.81675**	0.23329
I am afraid of having a bowel movement	0.15397	0.28777	**0.79261**	0.14189
I am uncomfortable after having a bowel movement	0.30312	0.08247	**0.67264**	0.23279
I have adapted my diet	0.21982	0.23674	**0.48396**	0.12746
I dread my next pregnancy	0.05788	0.17475	**0.33022**	‐0.04247
My sexual activity has decreased	0.24540	0.17766	0.18972	**0.85002**
I have sexual intercourse less often than I would like	0.26624	0.21252	0.14266	**0.84167**
My relations with my partner are disrupted	0.24811	0.23730	0.19480	**0.79236**
I feel less attractive	0.10828	0.45049	0.08913	**0.60139**
It affects my anal intercourse (bleeding, pain, etc.)	0.06525	0.01403	0.13813	**0.50363**
Variance	7.824	5.361	4.075	3.957

The highest value is reported in bold.

**Table 4:** List of questions removed from the questionnaire and reasons

Dimension	Questions	Reasons for removal
Physical disorders	I experience discomfort when I climb the stairs	− Similar coefficient to ‘I am uncomfortable while walking’ in the PCA. − ‘I am uncomfortable while walking’ encompasses better the walking activity
	I have to wear protection	− Very low coefficient in the PCA (0.343). − Deleted by BCAC
	I'm worried about staining my clothes or the armchair	− Only item dealing with bleeding. − Higher intensity in haemorrhoidal patients
Psychology	I get less out of life	− Similarity with ‘I feel uncomfortable with people around me’
	The pain, blood, and lumps worry me	− Too low a discriminant coefficient in the GRM (0.22)
	I feel tired and exhausted	− Slope below 1 in the GRM (0.83)
	My symptoms make me fear I have cancer (or a serious disease)	− This question brings little information for the dimension (0.52) (GRM)
	I find it hard to get to sleep	− Too low a slope for the dimension (0.80) (GRM)
	I feel as if nobody understands my situation	− Semantics issue. − Very similar to ‘I feel as if I am different from others’
Defaecating	I dread bowel movements	− Semantics issue. − ‘I am afraid of having a bowel movement’ measures the same concept and is easier to understand
	I have adapted my diet	− Usually, patients adapt their diet following recommendation from their physician and not on their own. − BCAC results
	I dread my next pregnancy	− This question concerns only women. − Very low coefficient in the PCA (0.330). BCAC results
Sexuality	I feel less attractive	− BCAC results
	I have sexual intercourse less often than I would like	− Very difficult to answer, ‘not applicable’ for this question. − ‘My sexual activity has decreased’ encompasses the evaluated concept better
	It affects my anal intercourse (bleeding, pain, etc.)	− Following BCAC, this question does not constitute a single dimension with others

BCAC, backward Cronbach alpha curves; PCA, principal component analysis; GRM, graded response model.

Based on the results of BCAC and GRM, respectively, two and four items were removed (Table 4), yielding a final questionnaire with 23 questions, each including six modalities (a five‐point Likert scale and the ‘not applicable’ modality). Indeed, for the modalities ‘often’ and ‘sometimes’, most *P*‐values were not significant in the graded response model. As a consequence, these modalities were clustered to create a new ‘regularly’ modality. Therefore, the questionnaire was reduced in terms of both the number of questions and the answer modalities.

The results of multi‐trait analysis (MTA) are shown in Table 5. The calculated correlation coefficients were high for the dimension to which each item belonged and low for the other dimensions, confirming the convergent and divergent validity of the questionnaire. These results contributed to validating the internal structure of the final version of the questionnaire.

**Table 5:** Results from the multi‐trait analysis

Dimensions	Items	Physical	Psychology	Defaecation	Sexuality	Total score
Physical disorders	Q1. It is uncomfortable to remain seated	**0.74731**	0.51957	0.44781	0.38472	0.70087
	Q3.It is uncomfortable to remain standing	**0.77325**	0.52657	0.43693	0.45220	0.72801
	Q5. I am uncomfortable while walking	**0.81677**	0.50785	0.40882	0.43878	0.73443
	Q20. I am uncomfortable when doing house chores/tidying up/handiwork	**0.83957**	0.54474	0.42076	0.39373	0.75589
	Q9. I am uncomfortable when I play sports	**0.82405**	0.48065	0.34840	0.39031	0.70706
	Q11. Driving a vehicle is difficult	**0.76763**	0.53779	0.46094	0.42052	0.72662
	Q13. Riding a two‐wheeled vehicle or bicycle is difficult	**0.82656**	0.56467	0.44602	0.44107	0.76474
	Q14. I find it difficult to do my work well	**0.79596**	0.66440	0.49866	0.44283	0.79539
	Q16. I do fewer things than I would want to do	**0.80005**	0.60824	0.48124	0.45608	0.77543
	Q18. I avoid going out (travel, leisure, friends, …)	**0.70438**	0.63304	0.41518	0.44085	0.71907
	Q2. I have to change clothes regularly or use a special type of clothing	**0.62259**	0.50119	0.32034	0.45583	0.61396
Psychology	Q19. My family life is disrupted	0.68895	**0.69958**	0.47883	0.59126	0.76824
	Q12. Taking care of my children is difficult	0.62932	**0.85657**	0.48253	0.59855	0.77021
	Q21. I am uncomfortable in my own body	0.55530	**0.72598**	0.49492	0.48215	0.67646
	Q8. I feel uncomfortable with people around me	0.53452	**0.68387**	0.38377	0.46055	0.62936
	Q15. I feel as if I am different from others	0.52172	**0.75806**	0.44298	0.41970	0.64701
	Q23. I believe that my illness is incurable	0.39824	**0.61308**	0.39847	0.39651	0.52332
	Q6. I feel ashamed	0.43013	**0.64227**	0.35242	0.39974	0.54231
Defaecation	Q10. I am uncomfortable during bowel movements	0.41676	0.45110	**0.80719**	0.38490	0.54522
	Q22. I am uncomfortable after having a bowel movement	0.50375	0.47042	**0.68806**	0.38567	0.58884
	Q7. I am afraid of having a bowel movement	0.45445	0.51466	**0.69954**	0.37739	0.57809
Sexuality	Q4. My relations with my partner are disrupted	0.51356	0.62161	0.42653	**0.85263**	0.63767
	Q17. My sexual activity has decreased	0.49759	0.54101	0.41145	**0.85263**	0.59876

The highest value is reported in bold.

Finally, as shown in Table 6, the HF‐QoL scores correlated strongly with those of the SF‐12 and PGWBI (*P *<* *0.001). These correlations were clinically consistent. Thus, the physical disorders dimension of the HF‐QoL questionnaire correlated strongly with the physical health dimension of SF‐12 and the general health dimension of the PGWBI. Similarly, the psychology dimension of the HF‐QoL questionnaire correlated strongly with the mental health score of SF‐12 and the global score of PGWBI. The dimensions defaecation and sexuality were more specific to the HF‐QoL questionnaire and consequently had weaker correlations with the SF‐12 and PGWBI.

**Table 6:** Spearman correlation coefficients between the HF‐QoL questionnaire, SF‐12 and the PGWBI

Dimensions	SF‐12^a^		PGWBI^a^						
	Physical health	Mental health	Anxiety	Depression	Well‐being	Control	General health	Vitality	Global
Physical disorders	*−0.586*	*−*0.417	*−*0.384	*−*0.363	*−*0.477	*−*0.448	*−0.609*	*−*0.466	*−0.560*
Psychology	*−*0.398	*−0.493*	*−*0.457	*−*0.501	*−*0.491	*−*0.501	*−0.524*	*−*0.436	*−0.594*
Defaecation	*−*0.297	*−*0.330	*−*0.332	*−*0.330	*−*0.274	*−*0.350	*−*0.498	*−*0.297	*−*0.425
Sexuality	*−*0.326	*−*0.344	*−*0.208	*−*0.327	*−*0.318	*−*0.341	*−*0.437	*−*0.283	*−*0.375
Total score	*−*0.557	*−*0.497	*−*0.461	*−*0.467	*−*0.511	*−*0.512	*−*0.651	*−*0.485	*−*0.628

a *P *<* *0.001 are reported in italic.

While no difference was found between haemorrhoids and anal fissures for the physical, psychology and sexuality scores, patients with anal fissures had much higher defaecation and total scores (Table 7).

**Table 7:** HF‐QoL scores per dimension depending on patient disease

		Haemorrhoidal disease	Anal fissure	Haemorrhoidal disease and anal fissure	Total
Physical disorders	*n*	171	75	9	255
	Mean	28.67	31.41	44.33	30.03
	SD	24.48	21.99	21.33	23.78
	Min.	0.00	0.00	9.38	0.00
	Median	25.00	30.56	47.22	28.13
	Max.	100.00	88.64	80.00	100.00
		*P*‐value (Kruskal–Wallis)			0.0720
Psychology	*n*	172	75	9	256
	Mean	21.54	24.89	32.46	22.90
	SD	19.87	21.75	19.06	20.47
	Min.	0.00	0.00	7.14	0.00
	Median	17.86	20.83	28.57	17.86
	Max.	89.29	85.71	60.00	89.29
		*P*‐value (Kruskal–Wallis)			0.1645
Defaecation	*n*	170	74	9	253
	Mean	44.03	66.72	73.15	51.70
	SD	28.46	25.07	20.74	29.35
	Min.	0.00	0.00	50.00	0.00
	Median	41.67	66.67	66.67	50.00
	Max.	100.00	100.00	100.00	100.00
		*P*‐value (Kruskal–Wallis)			<0.0001
Sexuality	*n*	170	75	9	254
	Mean	32.93	40.11	42.82	35.40
	SD	29.38	27.26	33.19	29.01
	Min.	0.00	0.00	0.00	0.00
	Median	35.40	50.00	37.50	35.40
	Max.	100.00	100.00	75.00	100.00
		*P*‐value (Kruskal–Wallis)			0.1124
Total score	*n*	169	74	9	252
	Mean	29.08	35.14	44.35	31.40
	SD	20.71	18.75	17.50	20.32
	Min.	0.00	3.26	16.44	0.00
	Median	26.45	32.90	45.98	30.08
	Max.	78.26	85.69	76.09	85.69
		*P*‐value (Kruskal–Wallis)			0.0079

### Reliability

Correlations between scores for all dimensions of the HF‐QoL questionnaire were significant (*P* < 0.001), and the CAC ranged from 0.86 to 0.95 for all items, reflecting the good internal consistency (>0.7) of the different dimensions.

### HF‐QoL scores according to clinical presentation

Table 8 shows that quality of life, based on the mean total HF‐QoL score, was more specifically impaired in patients with thrombosis (42.3), anal fissure (35.1), abundant anal bleeding (34.9) and high‐grade prolapse (31.5 for Grade IV).

**Table 8:** Total score of HF‐QoL according to disease presentation

	*N*	Total HF‐QoL score, mean (SD)	*P*‐value
Haemorrhoidal disease			
Anal bleeding	84	25.6 (20.0)	<0.0001^a^
Prolapse	40	21.5 (14.7)	
Thrombosis	45	42.3 (21.0)	
Total	169	29.1 (20.7)	
Prolapse grade			
Grade I	9	11.0 (12.1)	0.0052^a^
Grade II	43	19.9 (17.1)	
Grade III	58	27.9 (19.6)	
Grade IV	13	31.5 (15.5)	
Total	123	24.2 (18.6)	
Anal bleeding last week			
Abundant	24	34.9 (22.2)	0.0102^b^
Minimal	56	21.5 (18.2)	
Total	80	25.5 (20.3)	
Anal fissure only			
Acute	23	34.5 (17.0)	0.8414^c^
Chronic	51	35.4 (19.7)	
Total	74	35.1 (18.8)	

Results are given as mean (SD) of total HF‐QoL score.

a Kruskal–Wallis test.

b Wilcoxon test.

c Student's *t*‐test.

In the entire study population there was a significant correlation between the HF‐QoL total score and the VAS pain score (*r* = 0.560 for global pain and *r* = 0.589 for current pain, respectively; *P *<* *0.0001 for both).

A significant correlation between the pain score and the HF‐QoL total score was also observed in specific populations, namely patients with thrombosis (*r* = 0.441 for global pain and *r* = 0.435 for current pain; *P *<* *0.0025 and 0.0036, respectively) and patients with anal fissure only (*r* = 0.554 for global pain and *r* = 0.581 for current pain, respectively; *P* < 0.0001 for both).

### HF‐QoL total score and disease burden

Patients who were forced to take time off sick or to stop work because of their anal problems within the last 6 months had a significantly higher HF‐QoL total score (47.5 *vs* 29.3; *P* < 0.0001) (Table 9). Patients who reported that their anal problems had personal financial implications also had a significantly higher HF‐QoL total score (48.3 *vs* 27.0; *P *<* *0.0001).

**Table 9:** Total score of HF‐QoL and impact on work and personal finance

	*n*	Total HF‐QoL score, mean (SD)	*P*‐value^a^
Have you a professional activity?			
No	64	32.6 (22.4)	0.75
Yes	187	31.0 (19.7)	
Total	251	31.4 (30.4)	
Have your anal symptoms forced you to be off sick or stop working over the last 6 months?			
No	214	29.3 (19.4)	<0.0001
Yes	22	47.5 (18.6)	
Total	236	31.0 (20.0)	
Have your anal problems an impact on your personal budget?			
No	195	27.0 (18.4)	<0.0001
Yes	53	48.3 (18.3)	
Total	248	31.5 (20.3)	

a Wilcoxon test.

## Discussion

We describe the psychometric validation of the first questionnaire designed to quantify the global impact of haemorrhoidal disease and anal fissures on quality of life. The scores on the HF‐QoL questionnaire increased with symptom severity (pain, bleeding, prolapse) in patients with haemorrhoidal disease and anal fissures, and also with the impact on daily life (days off work, increased personal spending related to the disorders).

The very good and clinically consistent correlations of the physical disorders and psychology dimensions of the HF‐QoL questionnaire with the relevant dimensions of widely used and validated quality‐of‐life scales (SF‐12 and PGWBI) endorse the quality of the selected items to measure the impact on QoL. This is further reinforced by the weaker correlations between the disease‐specific dimensions of the HF‐QoL questionnaire (defaecation and sexuality) and the different dimensions of SF‐12 and PGWBI. Thus, this new tool quantifies both the general impact of these painful disorders and their specific impact on the affected body region (anus).

Significant correlations were obtained between the VAS pain scores and the HF‐QoL total score, both in the entire study population and also in subgroups of patients with haemorrhoidal thrombosis or anal fissure alone. Patients with anal fissures had higher mean subscores for the defaecation dimension, indicating a greater impact on quality of life than in patients with haemorrhoids. This is consistent with the significantly higher VAS pain scores in patients with fissures than in patients with haemorrhoidal disease. This is the first study to clearly show what is routinely observed by proctologists in clinical practice, namely the greater pain intensity and symptom burden of anal fissures relative to haemorrhoidal disease.

Only two questions were retained for the sexuality dimension, but removing this dimension would have prevented the assessment of this aspect of daily life, which also appears to be affected by these disorders.

Other tools have recently been developed to evaluate common proctological disorders, but they focus more on symptom severity than on quality of life. Thus, Kraemer *et al*. proposed a VAS‐based proctological symptom scale for pain, itching/irritation, discharge/moisture and bleeding, but excluded prolapse 19. Another statistically validated scoring system for haemorrhoidal disease was proposed by Pucher *et al*. 10, who assessed six symptoms (bleeding, pruritus, rest pain, defaecation pain, prolapse and soiling) in patients with haemorrhoidal disease and finally selected four (pruritus, rest pain, defaecation pain and prolapse).

Our study has some limitations. The decision to use a single questionnaire for both haemorrhoidal disease and anal fissures was taken *a priori*, based on the similarity of the answers given by the two groups of patients while developing the questionnaire. This decision is *post hoc* supported by our statistical analysis. The use of a single questionnaire is logical given the similar symptoms of these two conditions, namely pain, swelling (related to haemorrhoidal prolapse or skin tags associated with anal fissures), bleeding and pruritus. Another limitation is the lack of sensitivity of the analysis to clinical changes, based on score changes after treatment. This will be looked at in further studies. A test–retest measure of reliability has not been performed and temporal stability will require another study.

In conclusion, we have developed and validated a questionnaire designed to evaluate the burden of haemorrhoidal disease and anal fissures with respect to patients’ daily lives. The HF‐QoL questionnaire scores correlate with all features of these two disorders. This simple tool should prove useful for evaluation of the burden of illness in both clinical trials and in daily practice, in longitudinal and cross‐sectional designs, when the patient point of view needs to be disease‐specific, i.e. when a generic QoL instrument is not enough. This questionnaire is available in French and English.

## Study group

C. M. Eleouet‐Kaplan, Hôpital Bagatelle, Talence, France; D. Soudan, Groupe Hospitalier Saint Joseph, Paris, France; Alexandra Popescu, AP‐HP, Proctologie Médico‐Chirurgicale, Service de Gastroentérologie, CHU Bichat, Paris, France.

## Conflicts of interest

AZ and GB were employees of Pierre Fabre SA at the time of the writing of the manuscript; LA is a consultant for Abbvie, Allergan, Bayer, Coloplast, F Care, Meda and Sanofi. The other authors did not declare any conflict of interest.

## Supporting information


**Appendix S1.** HEMO‐FISS questionnaire.
