# Supplementary material for: Psychometric properties of a questionnaire (HEMO‐FISS‐QoL) to evaluate the burden associated with haemorrhoidal disease and anal fissures
Source: Colorectal Dis. 2018 Sep 21;21(1):48–58. doi: 10.1111/codi.14393 (PMC7379620; doi:10.1111/codi.14393)
Supplement: Supplementary file 1 — Appendix S1. HEMO‐FISS questionnaire. [file CODI-21-48-s001.docx]

Appendix

## HEMO-FISS Questionnaire

Please answer as spontaneously as possible. There are no right or wrong answers.

If you think that a question or statement does not apply to you, please check the box "Not applicable". For example: "I find it difficult to take care of my children because of my anal symptoms." If you do not have children, please check "Not applicable".

| **Last week,**  **because of my anal symptoms ...** | Always | Very  often | Regularly | Rarely | Never | Not  applicable |
| --- | --- | --- | --- | --- | --- | --- |
| Q1 ... it is uncomfortable to remain seated | 🖵 | 🖵 | 🖵 | 🖵 | 🖵 | 🖵 |
| Q2 ... I have to change clothes regularly or use a special type of clothing | 🖵 | 🖵 | 🖵 | 🖵 | 🖵 | 🖵 |
| Q3... it is uncomfortable to remain standing | 🖵 | 🖵 | 🖵 | 🖵 | 🖵 | 🖵 |
| Q4 ... my relations with my partner are disrupted | 🖵 | 🖵 | 🖵 | 🖵 | 🖵 | 🖵 |
| Q5 ... I am uncomfortable while walking | 🖵 | 🖵 | 🖵 | 🖵 | 🖵 | 🖵 |
| Q6 ... I feel ashamed | 🖵 | 🖵 | 🖵 | 🖵 | 🖵 | 🖵 |
| Q7 ... I am afraid of having a bowel movement | 🖵 | 🖵 | 🖵 | 🖵 | 🖵 | 🖵 |
| Q8 ... I feel uncomfortable with people around me | 🖵 | 🖵 | 🖵 | 🖵 | 🖵 | 🖵 |
| Q9 ... I am uncomfortable when I play sports | 🖵 | 🖵 | 🖵 | 🖵 | 🖵 | 🖵 |
| Q10 ... I am uncomfortable during bowel movements | 🖵 | 🖵 | 🖵 | 🖵 | 🖵 | 🖵 |
| Q11 ... driving a vehicle is difficult | 🖵 | 🖵 | 🖵 | 🖵 | 🖵 | 🖵 |
| Q12 ... taking care of my children is difficult | 🖵 | 🖵 | 🖵 | 🖵 | 🖵 | 🖵 |
| Q13 ... riding a two-wheeled vehicle or bicycle is difficult | 🖵 | 🖵 | 🖵 | 🖵 | 🖵 | 🖵 |
| Q14 ... I find it difficult to do my work well | 🖵 | 🖵 | 🖵 | 🖵 | 🖵 | 🖵 |
| Q15 ... I feel as if I am different from others | 🖵 | 🖵 | 🖵 | 🖵 | 🖵 | 🖵 |
| Q16 ... I do fewer things than I would want to do | 🖵 | 🖵 | 🖵 | 🖵 | 🖵 | 🖵 |
| Q17 ... my sexual activity has decreased | 🖵 | 🖵 | 🖵 | 🖵 | 🖵 | 🖵 |
| Q18 ... I avoid going out (travel, leisure, friends...) | 🖵 | 🖵 | 🖵 | 🖵 | 🖵 | 🖵 |
| Q19 ... my family life is disrupted | 🖵 | 🖵 | 🖵 | 🖵 | 🖵 | 🖵 |
| Q20 ... I am uncomfortable when doing house chores / tidying up / handy work | 🖵 | 🖵 | 🖵 | 🖵 | 🖵 | 🖵 |
| Q21 ... I am uncomfortable in my own body | 🖵 | 🖵 | 🖵 | 🖵 | 🖵 | 🖵 |
| Q22 ... I am uncomfortable after having a bowel movement | 🖵 | 🖵 | 🖵 | 🖵 | 🖵 | 🖵 |
| Q23 ... I believe that my illness is incurable | 🖵 | 🖵 | 🖵 | 🖵 | 🖵 | 🖵 |

**Coding algorithm**

This table will help clinicians to calculate the scores by completing it.

|  | **Physical disorders PD** | **Psychology P** | **Defecating D** | **Sexuality S** | **Overall*** |
| --- | --- | --- | --- | --- | --- |
| *Number of documented items (#1)* |  |  |  |  |  |
| *Number of items with documented severity, i.e. excluding not applicable (#2)* |  |  |  |  | Sum of PD, P, D and S |
| *Sum of items i.e. excluding not applicable [#3)* | Only if #1 > 5 | Only if #1 > 3 | Only if #1 > 1 | Only if #1 > 0 | Sum of PD, P, D and S |
| *Interim Score (#4)* | #3/#2 | #3/#2 | #3/#2 | #3/#2 | #3/#2 |
| *Final score* | (#4-1)/4*100 | (#4-1)/4*100 | (#4-1)/4*100 | (#4-1)/4*100 | (#4-1)/4*100 |

*Overall score is available if and only if all scores are estimated

Follow the hereunder steps:

1. Count of documented answered, “not applicable” being included
2. Count of documented answered, “not applicable” being excluded
3. An interim score is estimated if and only if at least 50% of the questions of the score are documented. Modality values to apply are the following: never =1, rarely = 2, regularly =3, very often =4, always =5.
4. Divide sum by sample size
5. Standardization to get a score between 0 and 100
